# l-Arginine deprivation impairs *Leishmania major*-specific T-cell responses

**DOI:** 10.1002/eji.200839041

**Published:** 2009-08

**Authors:** Markus Munder, Beak-San Choi, Matthew Rogers, Pascale Kropf

**Affiliations:** 1 Department of Hematology, Oncology, and Rheumatology, University Hospital HeidelbergHeidelberg, Germany; 2 Department of Immunology, Faculty of MedicineImperial College London, UK

**Keywords:** Cell activation, Cell proliferation, T cells, Th1/Th2 cells

## Abstract

The amino acid l-arginine plays a crucial role in the regulation of immune responses. We have recently shown that uncontrolled replication of *Leishmania* parasites at the site of pathology correlates with high levels of arginase activity in nonhealing leishmaniasis and that this elevated arginase activity causes local depletion of l-arginine. To further our understanding of the impact of l-arginine deprivation in experimental leishmaniasis, here we characterize in detail the effects of l-arginine deprivation on antigen-specific T cells and MΦ. The results of our study show that decrease of l-arginine levels in the extracellular milieu affects the biological activities of *Leishmania major*-specific T cells, both at the level of the magnitude and the quality of their responses. *L*. *major*-specific CD4^+^ T cells rendered hyporesponsive by l-arginine deprivation can be partially rescued by addition of exogenous l-arginine to produce IL-4 and IL-10, but not to produce IFN-γ. Furthermore, our results show that l-arginine deprivation also greatly impacts parasite growth in activated macrophages. In summary, our results suggest that l-arginine levels affect both Th cell responses and parasite replication.

## Introduction

The metabolism of the semi-essential amino acid l-arginine 1,2 by arginase is emerging as a crucial mechanism for the regulation of immune responses. Arginase 1 is upregulated in myeloid cells in response to a range of signals such as Th2 cytokines 3, GM-CSF 4,5, prostaglandin 6–8 and catecholamines 8. Arginase 1 has been shown to affect T-cell responses by reducing the availability of l-arginine: high arginase activity expressed by myeloid cells results in increased uptake of extracellular l-arginine into the cells, thereby reducing l-arginine levels in the microenvironment; this decrease in l-arginine results in T-cell hyporesponsiveness 9–13.

Experimental infection of mice with *Leishmania major* has been extensively characterized as a model for host resistance or susceptibility mediated by distinct Th subsets. The majority of inbred strains of mice develop small lesions that heal spontaneously within a few weeks, leaving the host immune to reinfection; this ability to control parasite replication correlates with the expansion of CD4^+^ Th1 cells, characterized by the production of IFN-γ. On the other hand, a few strains of mice such as BALB/c develop progressive nonhealing disease, attributed to the expansion of CD4^+^ Th2 cells, characterized by the production of IL-4, IL-10 and IL-13 14,15.

Macrophages (MΦ), the main host cells for the intracellular parasite *Leishmania*, are crucial for the outcome of disease: depending on the expression of two inducible enzymes, NO synthase 2 and arginase, MΦ can either kill the parasites or promote their growth. These two enzymes share a common substrate, l-arginine, and are competitively controlled by Th1 and Th2 cytokines 3,16. Th1 cytokines induce NO synthase 2 that oxidizes l-arginine into NO, a metabolite responsible for parasite killing 17,18. In contrast, Th2 cytokines result in the induction of arginase, which hydrolyzes l-arginine into ornithine, an amino acid that is the main intracellular source for the synthesis of polyamines; the latter are essential for parasite growth 19.

We have shown that uncontrolled replication of *Leishmania* parasites at the site of pathology correlates with high levels of arginase activity in nonhealing BALB/c mice, but not in healing CBA mice 19. This elevated arginase activity causes local depletion of l-arginine (P. Kropf, unpublished data). Therefore, in the present study we tested the effects of different levels of l-arginine on *Leishmania*-specific T-cell responses and MΦ effector functions.

## Results

### l-Arginine deprivation impairs antigen-specific T-cell effector functions

**Figure 1:**
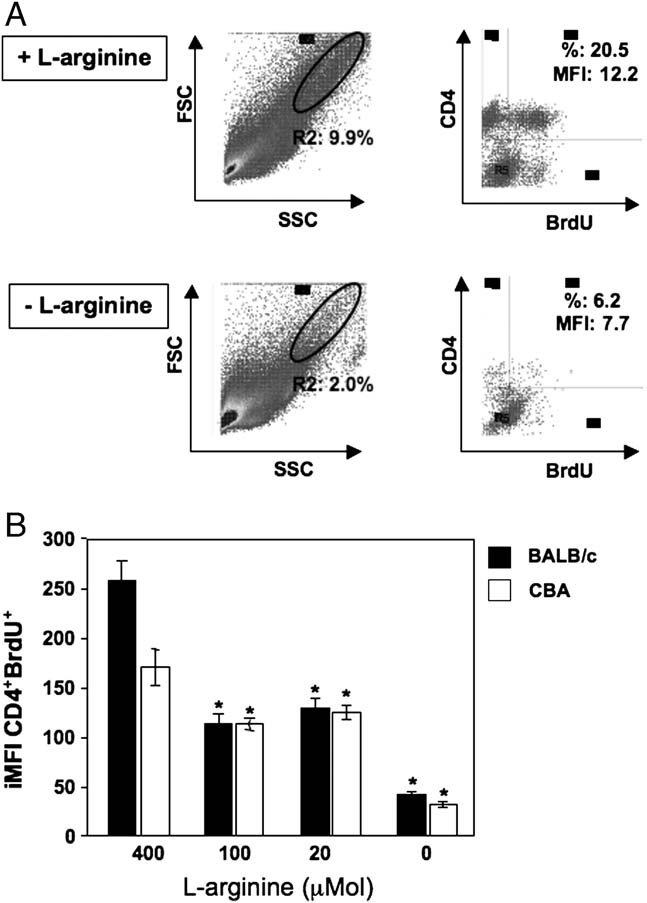
l-Arginine deprivation impairs CD4^+^ T-cell proliferation. Individual popliteal lymph nodes were harvested from BALB/c and CBA mice infected with *L*. *major* for 2 wk (*n*=4) and restimulated with *L*. *major* parasites in the presence (400 μM) or absence (0 μM) of l-arginine. After 5 days of *in vitro* restimulation, cells were harvested and the percentage of proliferating CD4^+^ T cells was determined as described in the *Materials and methods*. (A) Dot plot profiles of CD4^+^BrdU^+^ T cells (BALB/c mice); (B) iMFI of CD4^+^ BrdU^+^ T cells (BALB/c and CBA mice). Data show mean±SD of four individual lymph nodes/group. Isotype control: 0.59%. ^*^p<0.05 as determined by a two-tailed Mann–Whitney test. Data are representative of five independent experiments.

We and others have shown that polyclonal stimulation of T cells in the absence of l-arginine induces a profound T-cell hyporesponsiveness 10,12,13,20–23; however, little is known about the impact of l-arginine deprivation on antigen-specific responses. Here, we assessed the impact of l-arginine starvation on *L*. *major*-specific T-cell responses. To generate these cells, healer (CBA) and nonhealer (BALB/c) mice were infected with *L*. *major* parasites and 2 wk post infection, cells from popliteal lymph nodes were restimulated *in vitro* with *L*. *major* parasites 24. We first measured the proliferation of CD4^+^ T cells in response to antigenic restimulation in the presence (400 μM) or in the absence (0 μM) of l-arginine. The large majority (95.8%) of CD4^+^BrdU^+^ T cells from nonhealer BALB/c mice are found in R2 (Fig. 1A, upper left panel); this represents 9.9% of all gated cells. In contrast, in the absence of l-arginine, the percentage of proliferating CD4^+^ T cells is considerably lower (2.0%, Fig. 1A, lower left panel). In addition, l-arginine deprivation also induces a clear decrease in the frequency of proliferating CD4^+^ T cells (%CD4^+^BrdU^+^: 20.3±2.1% in the presence of l-arginine *versus* 2.1±0.3% in the absence of l-arginine, Fig. 1A). Similar results were obtained with CD4^+^ T cells from healer CBA mice (11.0±0.8% in the presence of l-arginine *versus* 2.8±0.1% in the absence of l-arginine, data not illustrated). A recent study has described a new metric parameter, the integrated MFI (iMFI), which reflects more precisely the total functional response of activated T cells 25. iMFI is calculated by multiplying the percentage, which represents the magnitude of the response by the MFI, which represents the quality of the response. As shown in Fig. 1B, there is a remarkable decrease in iMFI of CD4^+^BrdU^+^ in the absence of l-arginine (BALB/c: 392.2±32.6 *versus* 24.2±1.9, *p*<0.05; CBA: 133.1±11.8 *versus* 25.5±1.5, *p*<0.05), demonstrating that both the magnitude and the quality of the proliferative response of antigen-specific CD4^+^ T cells are greatly impaired in the absence of l-arginine.

We also assessed how different concentrations of l-arginine affect CD4^+^ T-cell effector functions. We used culture media containing 400, 100, 20 or 0 μM l-arginine and measured the proliferation of CD4^+^ T cells from *L*. *major*-infected nonhealer BALB/c and healer CBA mice. As shown in Fig. 1B, CD4^+^ T cells isolated from both healer and nonhealer mice proliferate less efficiently in response to antigen in the media containing 100 and 20 μM (*p*<0.05), and the sharpest reduction in proliferation was observed in the absence of l-arginine (0 μM, *p*<0.05).

**Figure 2:**
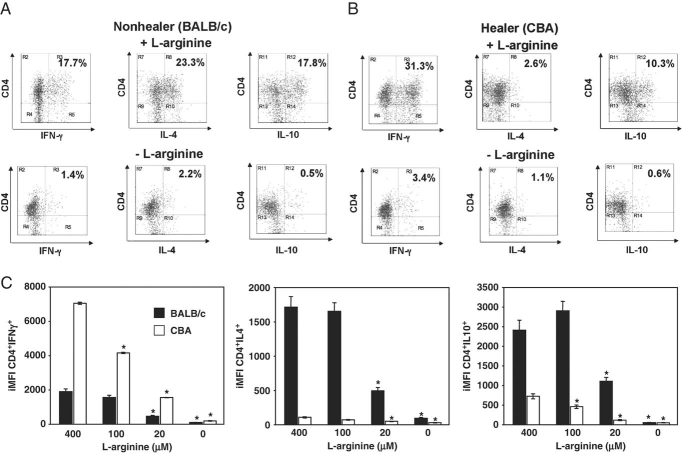
Impaired capacity of antigen-specific CD4^+^ T cells to express cytokines. Individual popliteal lymph nodes were harvested from BALB/c and CBA mice infected with *L*. *major* for 2 wk (*n*=4) and restimulated with *L*. *major* parasites in the presence (400 μM), absence (0 μM) of l-arginine (A and B) or at various concentrations of l-arginine (C). After 5 days of *in vitro* restimulation, cells were harvested and the percentage of cytokine-producing CD4^+^ T cells were determined as described in the *Materials and methods*. (A) Dot plot profiles of cytokine-producing CD4^+^ T cells (BALB/c mice; nonhealer). (B) Dot plot profiles of cytokine-producing CD4^+^ T cells (CBA mice; healer). (C) iMFI of cytokine-producing CD4^+^ T cells (BALB/c and CBA mice). Data show mean±SD of four individual lymph nodes/group. Isotype control for IFN-γ: 0.63±0.1%, IL-4: 0.78±0.12% and IL-10: 0.67±0.10%. ^*^p<0.05 as determined by a two-tailed Mann–Whitney test. Data are representative of five independent experiments.

To characterize further the impact of l-arginine deprivation on CD4^+^ T-cell effector functions, we assessed the ability of *L*. *major*-specific CD4^+^ T cells to produce IFN-γ, IL-4 and IL-10 in the absence of l-arginine. As shown in Fig. 2A and C, CD4^+^ T cells from nonhealing BALB/c mice display a strong polarized Th2-type response with high iMFI for CD4^+^IL-4^+^, but low iMFI for CD4^+^IFN-γ^+^; they also clearly produced the regulatory cytokine IL-10; in contrast, the cytokine profile of CD4^+^ T cells from healer CBA mice is clearly Th1-type, with high iMFI for IFN-γ and lower iMFI for IL-4 and IL-10 (Fig. 2B and C). Importantly, when cells from both groups of mice are restimulated with *L*. *major* parasites in the absence of l-arginine, there is a statistically significant reduction in the iMFI of CD4^+^IFN-γ^+^, CD4^+^IL-4^+^ and CD4^+^IL-10^+^ (*p*<0.05, Fig. 2A–C). Similar sharp reductions in the levels of IFN-γ, IL-4 and IL-10 were measured by Luminex in supernatants of restimulated lymph node cells from BALB/c and CBA mice (*p*<0.05, Table 1, Supporting Information).

**Figure 3:**
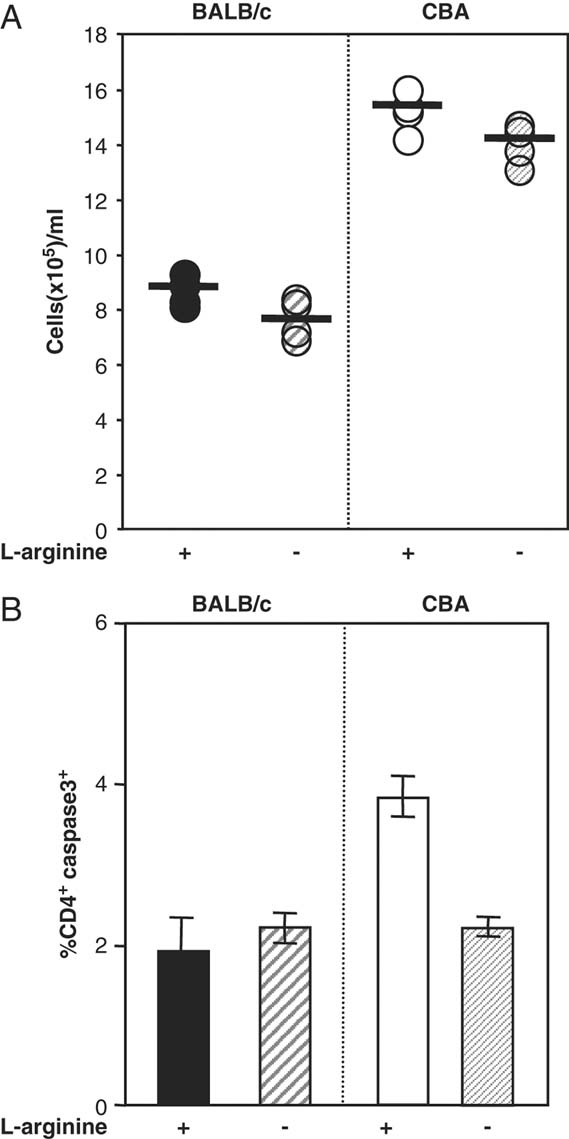
l-Arginine deprivation does not induce cell death. Individual popliteal lymph nodes were harvested from BALB/c and CBA mice infected with *L*. *major* for 2 wk (*n*=4) and restimulated with *L*. *major* parasites in the presence (400 μM) or absence (0 μM) of l-arginine. After 5 days of *in vitro* restimulation, cells were harvested, live cells were counted with Trypan blue staining (A) and the percentages of CD4^+^caspase3^+^ cells (B) were determined by flow cytometry. Data show mean±SD. Data are representative of three independent experiments.

We also measured the effects of different concentrations of l-arginine (400, 100, 20 or 0 μM l-arginine) on the antigen-specific cytokine production of CD4^+^ T cells: the iMFI of CD4^+^IFNγ^+^ T cells (CBA mice) diminishes steadily with lower concentration of l-arginine (Fig. 2C) and the iMFI for CD4^+^IL-4^+^ and CD4^+^IL-10^+^ T cells (BALB/c mice) remain unaltered at 400 and 100 μM, and start to decrease with lower concentrations of l-arginine (Fig. 2C).

l-Arginine deprivation did not induce increased cell death, as both the total cell number *per* culture (Fig. 3, upper panel) and the frequency of CD4^+^ caspase^+^ T cells (Fig. 3, lower panel) are similar in both the presence and absence of l-arginine, suggesting that cell death is not the cause for reduced T-cell activation and function in the absence of l-arginine.

### IL-4- and IL-10-producing CD4^+^ T cells can be partially rescued by addition of exogenous l-arginine

**Figure 4:**
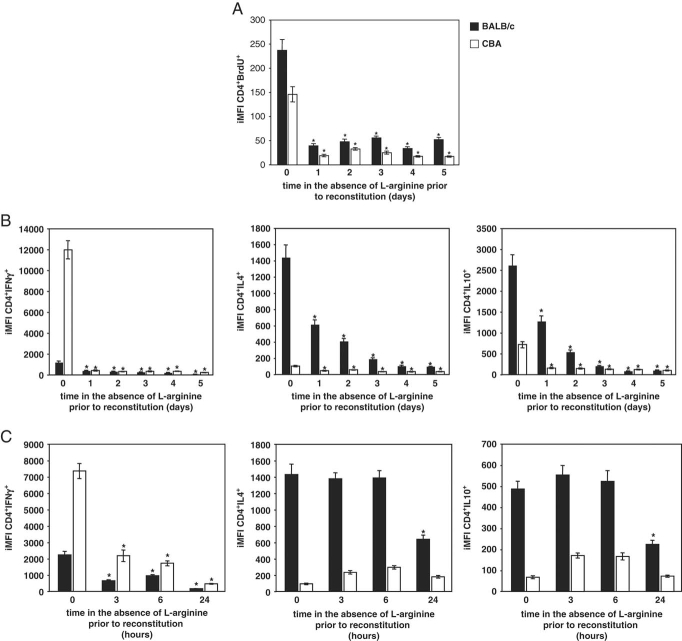
IL-4 and IL-10 production, but not IFN-γ production can be partially rescued by addition of exogenous l-arginine. Individual popliteal lymph nodes were harvested from BALB/c and CBA mice infected with *L*. *major* for 2 wk (*n*=4) and restimulated with *L*. *major* parasites in the absence of l-arginine. (A and B) After 1, 2, 3 or 4 days, 400 μM l-arginine was added to the cultures. In addition, some cells were restimulated with *L*. *major* parasites in the presence (400 μM, “0 day” group) or absence (0 μM, “5 day” group) of l-arginine. After 5 days, cells were harvested and the iMFI of CD4^+^BrdU^+^ cells (A) and the iMFI of cytokine-producing CD4^+^ T cells (B) were determined as described in the *Materials and methods*. (C) After 3, 6 or 24 h, l-arginine (400 μM) was added to the cultures. In addition, some cells were restimulated continuously with *L*. *major* parasites in the presence (400 μM, “0 hour” group) of l-arginine. After 5 days, cells were harvested and the iMFI of cytokine-producing CD4^+^ T cells were determined as described in the *Materials and methods*. Data show mean±SD of CD4^+^BrdU^+^ iMFI or IFN-γ^+^, IL-4^+^ or IL-10^+^ CD4^+^ iMFI from four individual lymph nodes/group. ^*^p<0.05 as determined by a two-tailed Mann–Whitney test. Data are representative of three independent experiments.

We have shown that stimulation of antigen-specific CD4^+^ T cells for 5 days in the absence of l-arginine results in severely impaired proliferation and cytokine production (Figs. 1 and 2). To determine whether CD4^+^ T cells rendered hyporesponsive by l-arginine deprivation could be rescued by addition of exogenous l-arginine, we stimulated lymph node cells from *L*. *major*-infected healer (CBA) and nonhealer (BALB/c) mice with *L*. *major* parasites as antigen in the absence of l-arginine (0 μM) and added l-arginine (400 μM) to the cultures after 1, 2, 3 or 4 days. As controls, cells from both strains of infected mice were restimulated with *L*. *major* parasites in the presence of l-arginine (400 μM) or in the absence of l-arginine (0 μM) for 5 days; as expected these cells display impaired proliferation in response to antigenic stimulation in the absence of l-arginine as compared with those in the presence of l-arginine (Fig. 4A). When exogenous l-arginine was added to the cells stimulated in the absence of this amino acid, proliferation of antigen-specific CD4^+^ T cells from both groups of mice could not be rescued, even when added as soon as 1 day post stimulation. Similarly, complementing the culture medium with 400 μM l-arginine after 1 day was not sufficient to induce CD4^+^ T cells to produce IFN-γ^+^ T cells (Fig. 4B). The inability to produce IFN-γ could even not be reversed by addition of l-arginine 3 h post restimulation (Fig. 4C). Th2 responses (BALB/c mice) could be partially rescued by addition of exogenous l-arginine after 1 and 2 days (Fig. 4B and C). Indeed, addition of l-arginine after 1 and 2 days still results in production of IL-4 (43 and 29% of total response) and IL-10 (49 and 21% of total response). To ensure that the l-arginine used to supplement the l-arginine-free medium was comparable to that of the commercially available DMEM, l-arginine-free DMEM was used and supplemented with 400 μM l-arginine; lymph nodes cells were resuspended in this medium, restimulated with *L*. *major* parasites and the proliferation and cytokine production was compared with those of cells restimulated in commercially available DMEM (400 μM). As presented in Table 2 (Supporting Information), the proliferation and cytokine production was similar in both conditions.

The results shown in Fig. 4B and C show that *L*. *major*-specific CD4^+^ T cells can be partially rescued to produce IL-4 and IL-10, but cannot be rescued to produce IFN-γ by addition of exogenous l-arginine *in vitro*.

**Figure 5:**
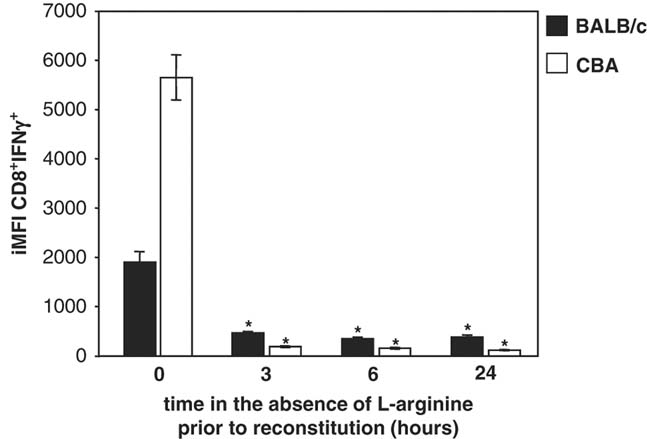
Impaired capacity of antigen-specific CD8^+^ T cells to express IFN-γ. Individual popliteal lymph nodes were harvested from BALB/c and CBA mice infected with *L*. *major* for 2 wk (*n*=4) and restimulated with *L*. *major* parasites in the absence of l-arginine. After 3, 6 or 24 h, l-arginine (400 μM) was added to the cultures. In addition, some cells were restimulated with *L*. *major* parasites in the presence (400 μM, “0 hour” group) of l-arginine. After 5 days, cells were harvested and the iMFI of cytokine-producing CD8^+^ T cells were determined as described in the *Materials and methods*. Data show mean±SD of IFN-γ^+^CD8^+^ iMFI from four individual lymph nodes/group. ^*^p<0.05 as determined by a two-tailed Mann–Whitney test. Data are representative of two independent experiments.

CD8^+^ T cells play an important role in immunity to *L*. *major* infection 26–29. Therefore, we next assessed the impact of l-arginine deprivation on *L*. *major*-specific CD8^+^ T cells. Similar to CD4^+^ T cells, CD8^+^ T cells from both groups of mice cannot proliferate in the absence of l-arginine (data not illustrated). We then measured their cytokine profile and show that CD8^+^ T cells from healer CBA mice produced more IFN-γ as compared with nonhealer BALB/c mice (Fig. 5); no IL-4 or IL-10 was detectable (data not illustrated). Similar to CD4^+^ T cells, antigen-specific CD8^+^ T cells cannot produce IFN-γ in response to antigenic restimulation in the absence of l-arginine and cannot be rescued by addition of exogenous l-arginine (Fig. 5, ^*^p<0.05).

### l-Arginine deprivation affects parasite growth in MΦ

**Figure 6:**
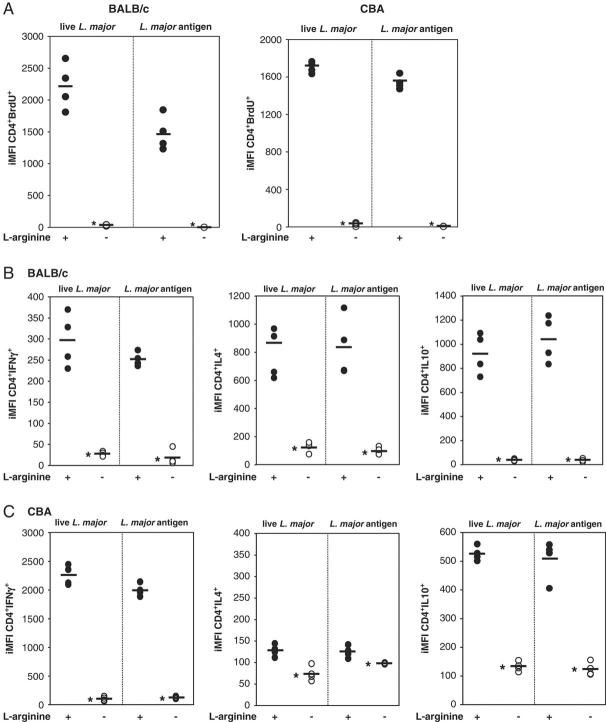
Impaired capacity of CD4^+^ T cells to proliferate and express cytokines in response to *L. major* antigen preparation. Individual popliteal lymph nodes were harvested from BALB/c and CBA mice infected with *L*. *major* for 2 wk (*n*=4) and restimulated with live *L*. *major* parasites or *L*. *major* antigen in the presence (400 μM) or absence (0 μM) of l-arginine. After 5 days, cells were harvested and percentage of CD4^+^BrdU^+^ T cells (A) and cytokine-producing CD4^+^ T cells isolated from BALB/c (B) or CBA (C) were determined by flow cytometry. Each symbol represents the value for BrdU^+^, IFN-γ^+^, IL-4^+^ or IL-10^+^ CD4^+^ iMFI of one individual lymph node/group with the horizontal bar as the mean value. ^*^p<0.05 as determined by a two-tailed Mann–Whitney test. Data are representative of two independent experiments.

It cannot be excluded that in the experiments described in Figs. 1–5, antigen presentation is also altered by the absence of l-arginine and therefore impacts on T-cell responses as well. It is not possible to use co-culture experiments with *L*. *major*-infected BMMΦ and T cells to dissect the impact of l-arginine deprivation on these two cell types, as we already know that lack of l-arginine results in impaired T-cell functions 10,12,13,20–23. Therefore, here we tested how l-arginine deprivation impacts on parasite growth and MΦ effector functions. *L*. *major* promastigotes growth in cultures was not affected by the absence of l-arginine: after 4 days in culture in the absence of l-arginine, the number of viable parasite grown in the absence of l-arginine was similar as that in the presence of l-arginine (211×10^6^±22×10^6^ *versus* 234×10^6^±12×10^6^, *p*>0.05). Moreover, the viability of the parasite did not seem to be critical as the use of *L*. *major* antigen preparation to restimulate lymphoid cells from BALB/c mice in the absence of l-arginine also resulted in a considerable reduction of both the proliferation (Fig. 6A, left panel, *p*<0.05) and cytokine production (Fig. 6B, left panel, *p*<0.05). Similar results were obtained with cells from CBA mice (Fig. 6A, right panel and Fig. 6C, *p*<0.05).

**Figure 7:**
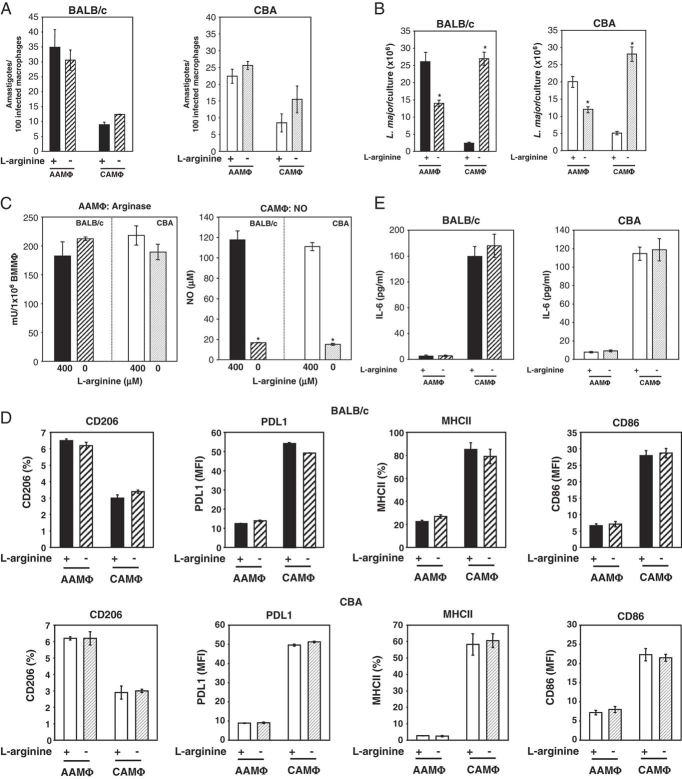
l-Arginine deprivation impairs parasite growth in activated MΦ. Mature BMMΦ were differentiated into CAMΦ or AAMΦ in the presence (400 μM) or in the absence (0 μM) of l-arginine. After 18 h, MΦ were infected with *L*. *major* parasites. (A) After 4 h of infection, the capacity of activated MΦ to phagocytose *L*. *major* promastigotes was determined. The parasite growth in activated MΦ was measured after 2 days (B) and the arginase activity in AAMΦ (C, left panel) and NO production (C, right panel) were determined as described in the *Materials and methods*. (D) Expression of activation markers as assessed by flow cytometry. (E) Production of IL-6 by CAMΦ or AAMΦ. Data show mean±SD of four individual wells/group. ^*^p<0.05 as determined by a two-tailed Mann–Whitney test. Data are representative of two independent experiments.

Next, we assessed whether the capacity of activated MΦ to phagocytose live *L*. *major* parasites was affected by the absence of MΦ (Fig. 7B, *p*<0.05); however, in the absence of l-arginine, *L*. *major* parasites survived more efficiently than in the presence of l-arginine (*p*<0.05). Since parasite killing depends on the production of NO resulting from the catabolism of l-arginine by iNOS 17–19, we measured the production of NO by CAMΦ in the presence or absence of l-arginine and as expected, there was a drastic reduction in the production of NO in the absence of l-arginine (Fig. 7C, *p*<0.05).

To characterize the impact of l-arginine deprivation on MΦ activation, we analyzed the expression levels of activation markers on AAMΦ and CAMΦ. As shown in Fig. 7D, the absence of l-arginine during the differentiation of mature MΦ into CAMΦ and AAMΦ did not significantly affect the expression of CD206, PDL1, MHCII and CD86 (*p*>0.05). CD80 and CD69 were not upregulated in any of the MΦ subsets tested (data not shown). Since DC also play an important role in *Leishmania* infection, we assessed the impact of l-arginine deprivation on the activation of DC. Similar to BMMΦ, the expression levels of activation markers on DC activated with either IL-4 or IFN-γ and TNF-α were not affected by the absence of l-arginine (Table 3, Supporting Information). In the next step, we assessed whether activated *L*. *major*-infected DC and MΦ require l-arginine for the production of cytokines. Although no IL-10 or IL-12p70 were detectable under those conditions (data not shown), the levels of IL-6 produced by DC derived from both BALB/c and CBA mice in response to IFN-γ and TNF-α were not affected by l-arginine deprivation (*p*>0.05, Table 4, Supporting Information). Similarly, AAMΦ and CAMΦ derived from both BALB/c and CBA mice produce similar levels of IL-6 (*p*>0.05, Fig. 7E).

The results shown in Fig. 7 suggest that although MΦ effector functions tested here do not seem to be altered by l-arginine deprivation, the growth of *L*. *major* parasites in MΦ is affected by the absence of l-arginine.

## Discussion

Using experimental infection of mice from healer and nonhealer strains, we and others have previously shown that high arginase activity is a hallmark of nonhealing leishmaniasis 19,30,31. High enzymatic arginase activity requires high substrate turnover and indeed, high arginase activity enhances l-arginine catabolism *in vivo* resulting in significantly reduced levels of l-arginine (P. Kropf, unpublished data). In the present study, we characterized in detail the effects of l-arginine deprivation on the effector functions of *L*. *major*-specific T cells isolated from healer and nonhealer mice. The results of our study show that decrease of l-arginine levels in the extracellular environment affect the biological activities of *L*. *major*-specific T cells. These results are mainly based on the evaluation of the total functional response of antigen-specific T cells using a recently described metric parameter, the iMFI 25. Both the magnitude and quality of the proliferative responses of antigen-specific T cells from *L*. *major*-infected healer and nonhealer strains of mice are greatly impaired by l-arginine starvation.

In agreement with the results of Rodriguez *et al*. 22, we found here that increased cell death can be excluded as a cause for the impaired proliferative response of antigen-specific T cells. Our results suggest that cytokine production might be differentially affected by variable l-arginine levels: IFN-γ production by T cells decline very rapidly to decreasing l-arginine levels although IL-4- and IL-10-producing T cells maintained their responsiveness longer and tolerated decreasing l-arginine levels more efficiently. Of note, addition of exogenous l-arginine did not restore impaired IFN-γ production of hyporesponsive CD4^+^ T cells; however, it partially rescued IL-4 and IL-10 production. These results show that fluctuations in l-arginine levels might affect Th1 responses faster and more profoundly than Th2 responses. This differential responsiveness to decreasing l-arginine levels may contribute to the different manifestations of leishmaniasis, a disease requiring Th1-mediated immune responses for parasite killing and healing.

l-Arginine is essential for the efficient activation and function of T cells; depletion of this amino acid in the extracellular microenvironment by transport into myeloid cells *in vivo* – or modeled *in vitro* using l-arginine-free culture medium – induces profound T-cell hyporesponsiveness, as shown by downregulation of proliferation, cytokine production and CD3ζ chain expression 11–13,21,22,32. This T-cell dysfunction is directly attributed to l-arginine deprivation that controls the cell cycle and arrests the cells in the G0-G1 phase 33. High arginase expression has been associated with a variety of diseases 12,34–37; however, a direct causal relationship between high arginase activity, low l-arginine levels and T-cell hyporesponsiveness has only been shown in a few conditions such as cancer 38, tuberculosis 39 and pregnancy 13.

The effects of l-arginine depletion have been mainly characterized using polyclonally stimulated T cells 11–13,21–23,32; however, its effect on antigen-specific T cells has not been analyzed in detail. Some studies have taken advantage of OT-1 and OT-2 mice, which have one transgenic T-cell receptor specific for an ovalbumin-immunodominant peptide; more than 90% of T cells express the transgene, providing a large pool of T cells with one specificity. Stimulation of T cells from these mice with the relevant peptide in the absence of l-arginine resulted in strongly impaired T-cell responses 40,41.

We have recently shown that although l-arginine is unconditionally required for T-cell activation, MΦ can upregulate activation markers and produce cytokines and chemokines in the absence of l-arginine; moreover, the absence of l-arginine did not affect the capacity of activated MΦ to upregulate l-arginine-metabolizing enzymes such as inducible NO synthase and arginase 1 23. Here, we characterized the effects of l-arginine deprivation on *L*. *major*-infected AAMΦ and CAMΦ and show that it did not impair the phagocytosis of promastigotes. However, the growth of *L*. *major* parasite was greatly impaired in AAMΦ, which usually efficiently promote parasite growth 19,42. We have previously shown that parasite replication depends on the production of polyamines, which are derived from the catabolism of l-arginine by arginase; therefore, in the absence of l-arginine, the production of polyamine, and thereby the parasite growth, will be greatly impaired 19. Moreover, we also show here that in the absence of l-arginine, parasites can replicate more efficiently in CAMΦ, because of the low NO production in the absence of l-arginine. Although these experiments do not reveal whether antigen presentation is altered in the absence of l-arginine, these results show that l-arginine levels greatly affect parasite growth in activated MΦ. Importantly, the levels of antigen have been shown to determine the Th phenotype in experimental leishmaniasis: low-dose infection are characterized by a polarized Th1 response and healing and immunity to reinfection 43,44. Therefore, it is tempting to speculate that high arginase observed in nonhealing experimental leishmaniasis favors parasite growth and thereby also promotes a strong polarized Th2 response.

In summary, we have shown that l-arginine depletion strongly affects T-cell subsets induced by *L*. *major* infection. More detailed studies of the mechanisms resulting in suppression of T-cell responses by fluctuating l-arginine levels and identification of mechanisms as to how to restore them are likely to lead to new therapeutic or prophylactic means to treat not only leishmaniasis, but also diseases such as asthma and cancer.

## Materials and methods

### Mice

Six- to eight-week-old female BALB/c and CBA mice (Charles River, UK) were kept in individually vented cages. Animal colonies, screened regularly for mouse pathogens, consistently tested negative. Animal experiments were performed in accordance with Home Office and institutional guidelines.

### Experimental infection with *L*. *major* parasites

For infections, 2×10^6^ stationary phase *L*. *major* LV39 (MRHO/SU/59/P-strain) promastigotes were injected s.c. into the footpad 45.

### Flow cytometric analyses

Popliteal lymph nodes from 2 wk-infected BALB/c and CBA mice were homogenized and 5×10^6^/mL cells were activated with 1×10^6^ live *L*. *major* parasites or 1×10^6^ *L*. *major* antigen preparation (*L*. *major* frozen and thawed three times) in DMEM (400 μM l-arginine), graded concentrations of l-arginine (100 and 20 μM) or l-arginine-free DMEM (0 μM l-arginine), supplemented with 5% FBS, 50 IU/mL penicillin, 50 mg/mL streptomycin and 292 mg/mL l-glutamine (Gibco). Cells were harvested after 5 days for further analysis 24.

In the experiments where l-arginine was added to the cultures, a stock solution of 100 mM l-arginine (l-arginine-monohydrochlorid, Roth) was prepared and 400 μM was added to the cultures.

### Proliferation assay

Before surface labeling with anti-CD4 mAb (clone H129.19 or GK1.5, Pharmingen) or anti-CD8 mAb (Clone 53–6.7, eBioscience), cells were preincubated with 1 μg of rat anti-mouse mAb CD32/CD16 (FcγII/III receptor, Pharmingen). Cells were washed, fixed and permeabilized using the method described in 29. Detection of CD4^+^BrdU^+^ cells was performed using a FACSCalibur (Becton Dickinson) and data were analyzed using Summit v4.3 software.

### Intracellular cytokine determination

Cells (1×10^6^) were stimulated with 50 ng of PMA (Sigma) and 500 ng of ionomycin (Calbiochem) or, as a control, in the presence of complete medium alone for 4 h, with 10 μg of brefeldin A (Sigma) added for the last 2 h. Before surface labeling with anti-CD4 mAb (clone H129.19 or GK1.5, Pharmingen) or anti-CD8 mAb (Clone 53–6.7, eBioscience), cells were preincubated with 1 μg of rat anti-mouse mAb CD32/CD16 (FcγII/III receptor, Pharmingen). Cells were washed, fixed and permeabilized as described in 24 before the anti-cytokine antibodies or the isotype controls were added (anti-IL-4 mAb, clone BVD4-1D11; anti-IFN-γ mAb, clone XMG1.2; anti-IL-10 mAb, clone JES5-16E3; appropriately labeled rat immunoglobulin (Pharmingen)). Detection of intracellular cytokines was performed using a FACSCalibur (Becton Dickinson) and data were analyzed using Summit v4.3 software.

### iMFI

The iMFI 25 was obtained by multiplying the percentage of CD4^+^ or CD8^+^ T cells with the value of the MFI for BrdU or with the value of the MFI for the relevant cytokine.

### Activation markers

Anti-CD69, anti-MHCII, anti-CD86, anti-CD80, anti-CD69, anti-PDL1 (eBioscience) and anti-CD206 (Serotec) were used according to the supplier's protocols. Detection of activation markers was performed on a FACSCalibur (Becton Dickinson) and data were analyzed using the Summit 4.0 software.

### BMMΦ

BM was obtained by flushing the femurs of BALB/c and CBA mice and precursor cells were cultured in bacteria plates in DMEM containing 10% heat-inactivated FBS, 5% horse serum and the supernatant of L929 fibroblasts at a final concentration of 10% v/v as a source of CSF that drive the cell proliferation toward a pure population of BM-derived MΦ. After 7 days in culture, mature BMMΦ were harvested, activated for 2 days and infected with *L*. *majo*r parasites at a ratio of 5:1 in the presence (400 μM) or in the absence (0 μM) of l-arginine. To obtain CAMΦ, BMMΦ were stimulated with 100 U/ml IFN-γ (PeproTech) and 500 U/mL TNF-α (PeproTech); to obtain alternatively activated MΦAAMΦ, BMMΦ were stimulated with 20 U/ml IL-4 (PeproTech).

### Phagocytosis assay

BMMΦ were seeded into 16-well glass slides (Lab-Tek, Nunc) at a density of 7×10^4^ BMMΦ *per* well and activated as described in the section *BMMΦ*. Eighteen hours later, BMMΦ were infected at a multiplicity of infection of 5 *L*. *major* promastigotes to 1 MΦ (MOI 5:1) in a 37°C, 5% CO2 humidified incubator. Four hours later, the MΦ were washed four times with PBS to remove non-phagocytosed promastigotes. The slides were air dried, fixed in methanol for 5 min and stained in 10% v/v Giemsa solution for 10 min. The average intracellular parasitemia was determined by oil-immersion microscopy of at least 200 MΦ *per* treatment (performed in duplicate), using the formula: (♯parasites/♯infected cells) × (♯infected cells/total ♯cells) × 100. Infections are expressed as the average number of intracellular parasites *per* 100 infected MΦ.

### Parasite growth

A total of 5×10^5^ BMMΦ were activated as described above in a final volume of 1 mL; 18 h later, 25×10^5^ *L*. *major* parasite were added to the cultures and 2 days later, the plates were washed with PBS to remove non-phagocytosed promastigotes and the MΦ were lysed as described in 45. An aliquot of 100 μL of this suspension was added to 900 μL of Schneider's medium (Invitrogen, containing 10% FBS, 50 IU/mL penicillin, 50 mg/mL streptomycin and 292 mg/ml l-glutamine) and 6 days later, the number of parasite *per* culture was counted.

### DC

DC were generated as described in 3. Briefly, BM was obtained by flushing the femurs of BALB/c and CBA mice and precursor cells were cultured in medium containing 5 ng/mL GM-CSF for 10 days. DC were harvested, infected with *L*. *majo*r parasites at a ratio of 5:1 and activated for two days with either a combination of 100 U/mL IFN-γ (PeproTech) and 500 U/mL TNF-α (PeproTech) or with 20 U/mL IL-4 (PeproTech) in the presence (400 μM) or in the absence (0 μM) of l-arginine.

### Determination of arginase activity

Arginase activity was measured in MΦ lysates by the conversion of l-arginine to urea as described in 3,19. One unit of enzyme activity was defined as the amount of enzyme that catalyzes the formation of 1 μmol of urea *per* min.

### Nitrite determination

NO_2_^−^ accumulation was used as an indicator of NO production and measured using Griess reagent 45.

### Luminex

Lymphocytes, BMMΦ and DC were stimulated as described above; supernatants were harvested and frozen until further use. IFN-γ, IL-4 and IL-10 (lymphocytes) or IL-12p70, IL-10 and IL-6 (BMMΦ) were detected simultaneously in each sample by the Luminex-based Multiplexed assay (Luminex 100 System). Data were analyzed using STarstation V2.0.

### Statistical analyses

The reduction in responses observed between the results obtained in the presence as compared with those obtained in the absence of l-arginine were analyzed for statistical differences using a two-tailed Mann–Whitney test and differences were considered statistically significant at *p*<0.05.

## Abbreviations

AAMΦ: alternatively activated MΦ
CAMΦ: classically activated MΦ
iMFI: integrated MFI
