# Supplementary material for: l-Arginine deprivation impairs Leishmania major-specific T-cell responses
Source: Eur J Immunol. 2009 Aug;39(8):2161–72. doi: 10.1002/eji.200839041 (PMC2948424; doi:10.1002/eji.200839041)
Supplement: Supplementary file 1 [file eji0039-2161-SD1.pdf]

European Journal of Immunology

Supporting Information

for

DOI 10.1002/eji.200939041

L-arginine deprivation impairs *Leishmania major*-specific T-cell responses

Markus Munder, Beak-San Choi, Matthew Rogers and Pascale Kropf

© 2009 WILEY-VCH Verlag GmbH & Co. KGaA, Weinheim

<u>Table 1.</u> Antigen-specific cytokine production is reduced in the absence of L–arginine

**% reduction in the absence of L-arginine**	**IFN-γ**	**IL-4**	**IL-10**
**BALB/c**	86.5%	83.8%	97.4%
**CBA**	97.2%	95.4%	97.5%

Individual popliteal lymph nodes were harvested from two weeks *L. major* infected BALB/c and CBA mice (n=4) and restimulated with *L. major* parasites in the presence (400μM) or absence (0μM) of L-arginine. Three days later, supernatants were harvested and tested for their content of IFN-γ, IL-4 and IL-10 by Luminex.

Data show the results of one representative experiment out of two independent experiments.

<u>Table 2.</u> Antigen-specific cytokine production and proliferation are impaired in the absence of L–arginine

**Mice**	**Medium**	**iMFI CD4$^+$ BrdU$^+$**
BALB/c	DMEM (400μM)	221.5±36.5
BALB/c	L-arginine-free DMEM + 400μM L-arginine	245.9±26.9
BALB/c	L-arginine-free DMEM	21±1.2
CBA	DMEM (400μM)	121.3±11.2
CBA	L-arginine-free DMEM + 400μM L-arginine	121.3±18.2
CBA	L-arginine-free DMEM	12±3.2

**Mice**	**Medium**	**iMFI CD4$^+$ IFN-γ$^+$**
BALB/c	DMEM (400μM)	2111.2±145.2
BALB/c	L-arginine-free DMEM + 400μM L-arginine	1885.3±211.0
BALB/c	L-arginine-free DMEM	88.1±7.4
CBA	DMEM (400μM)	12003.9±1450.1
CBA	L-arginine-free DMEM + 400μM L-arginine	14210.3±1102.0
CBA	L-arginine-free DMEM	127±11.2

**Mice**	**Medium**	**iMFI CD4$^+$ IL-4$^+$**
BALB/c	DMEM (400μM)	1523.9±112.5
BALB/c	L-arginine-free DMEM + 400μM L-arginine	1621.1±123.5
BALB/c	L-arginine-free DMEM	102.8±4.6
CBA	DMEM (400μM)	51.7±2.0
CBA	L-arginine-free DMEM + 400μM L-arginine	45.6±4.5
CBA	L-arginine-free DMEM	2.8±0.2

**Mice**	**Medium**	**iMFI CD4$^+$ IL-10$^+$**
BALB/c	DMEM (400μM)	3210.0±352.1
BALB/c	L-arginine-free DMEM + 400μM L-arginine	3006.1±214.5
BALB/c	L-arginine-free DMEM	146.2±11.9
CBA	DMEM (400μM)	723.8±84.4
CBA	L-arginine-free DMEM + 400μM L-arginine	631.7±63.3
CBA	L-arginine-free DMEM	3.8±0.2

Individual popliteal lymph nodes were harvested from two weeks *L. major* infected BALB/c and CBA mice (n=4) and restimulated with *L. major* parasites in commercially available DMEM (400μM L-arginine), in L-arginine free DMEM supplemented with 400μM L-arginine or in L-arginine free DMEM. Five days later, cells were harvested and the iMFI of CD4$^+$BrdU$^+$ cells and the iMFI of cytokine-producing CD4$^+$ T cells were determined as described in Material and Methods. The

results represents the average for $BrdU^+$, IFN-γ^+, IL-4^+ or IL-10^+ $CD4^+$ iMFI of one individual lymph node/group and are ± standard deviations and data show the results of one representative experiment out of two independent experiments.

<u>Table 3.</u> Absence of L-arginine does not affect the expression levels of activation markers on dendritic cells

Stimulation of DC		IL-4		IFN-γ + TNF-α	
	L-arginine	+	-	+	-
MHCII (%)	BALB/c	5.1 ± 0.2	8.2 ± 0.9	29.5 ± 0.8	27.5 ± 1.4
	CBA	18.1 ± 0.8	16.8 ± 0.6	50.1 ± 1.6	48.2 ± 1.1
CD80 (%)	BALB/c	3.4 ± 0.4	6.9 ± 0.9	30.6 ± 0.8	26.3 ± 1.3
	CBA	3.9 ± 0.4	3.6 ± 0.5	17.4 ± 1.6	15.5 ± 1.4
CD86 (%)	BALB/c	3.7 ± 0.3	7.5 ± 0.1	30.7 ± 0.8	28.8 ± 3.4
	CBA	16.4 ± 1.7	16.3 ± 0.9	57.7 ± 1.3	56.0 ± 5.1
CD69 (%)	BALB/c	8.2 ± 1.4	7.9 ± 0.3	86.1 ± 0.7	89.6 ± 4.0
	CBA	8.7 ± 0.8	7.9 ± 0.3	93.5 ± 3.3	89.6 ± 1.5
CD54 (%)	BALB/c	8.2 ± 1.4	6.5 ± 1.4	86.1 ± 0.7	94.4 ± 4.0
	CBA	8.2 ± 0.8	8.1 ± 0.3	93.5 ± 3.3	89.6 ± 1.5
PDL1 (MFI)	BALB/c	92.5 ± 1.6	92.5 ± 1.8	87.4 ± 3.5	93.9 ± 0.2
	CBA	88.8 ± 0.6	83.2 ± 2.6	94.7 ± 3.0	93.1 ± 0.9

Dendritic cells were activated with IL-4 or IFN-γ and TNF-α as described in Materials and Methods in the presence (+ = 400μM) or in the absence (- = 0μM) of L-arginine, infected with *L. major* parasites and two days later, the expression levels of activation markers were determined by FACS. Each value is the average of four individual wells/group and is ± standard deviation. Data show the results of one representative experiment out of two independent experiments.

Table 4

Stimulation of DC		IL-4		IFN-γ + TNF-α	
	L-arginine	+	-	+	-
IL-6 (pg/ml)	BALB/c	108.7 ± 7.1	104.9 ± 6.1	789.6 ± 2.9	772.3 ± 55.5
	CBA	145.1 ± 10.6	140.2 ± 1.6	741.9 ± 39.0	816.5 ± 136.0

Dendritic cells were activated with IL-4 or IFN-γ and TNF-α as described in Materials and Methods in the presence (400μM) or in the absence (0μM) of L-arginine, infected with *L. major* parasites and two days later, supernatants were harvested and tested for the presence of cytokines by Luminex. Each value is the average of four individual wells/group and are ± standard deviation. Data show the results of one representative experiment out of two independent experiments.
